# Cerebrospinal fluid in the differential diagnosis of Alzheimer’s disease: clinical utility of an extended panel of biomarkers in a specialist cognitive clinic

**DOI:** 10.1186/s13195-018-0361-3

**Published:** 2018-03-20

**Authors:** Ross W. Paterson, Catherine F. Slattery, Teresa Poole, Jennifer M. Nicholas, Nadia K. Magdalinou, Jamie Toombs, Miles D. Chapman, Michael P. Lunn, Amanda J. Heslegrave, Martha S Foiani, Philip S. J. Weston, Ashvini Keshavan, Jonathan D. Rohrer, Martin N. Rossor, Jason D. Warren, Catherine J. Mummery, Kaj Blennow, Nick C. Fox, Henrik Zetterberg, Jonathan M. Schott

**Affiliations:** 1 Dementia Research Centre, UCL Institute of Neurology, 8–11 Queen Square, London, WC1N 3BG UK; 2 Lila Weston Institute, UCL Institute of Neurology, London, UK; 3 Department of Medical Statistics, London School of Hygiene & Tropical Medicine, London, UK; 4 Department of Molecular Neuroscience, Institute of Neurology, UCL, London, UK; 5 Department of Neuroimmunology, National Hospital for Neurology and Neurosurgery, Queen Square, London, UK; 6 Department of Psychiatry and Neurochemistry, Institute of Neuroscience and Physiology, The Sahlgrenska Academy at University of Gothenburg, Sahlgrenska University Hospital, Mölndal, Sweden; 7 Clinical Neurochemistry Laboratory, Sahlgrenska University Hospital, Mölndal, Sweden

**Keywords:** Cerebrospinal fluid, Biomarkers, Alzheimer’s disease, Differential diagnosis

## Abstract

**Background:**

Cerebrospinal fluid (CSF) biomarkers are increasingly being used to support a diagnosis of Alzheimer’s disease (AD). Their clinical utility for differentiating AD from non-AD neurodegenerative dementias, such as dementia with Lewy bodies (DLB) or frontotemporal dementia (FTD), is less well established. We aimed to determine the diagnostic utility of an extended panel of CSF biomarkers to differentiate AD from a range of other neurodegenerative dementias.

**Methods:**

We used immunoassays to measure conventional CSF markers of amyloid and tau pathology (amyloid beta (Aβ)1–42, total tau (T-tau), and phosphorylated tau (P-tau)) as well as amyloid processing (AβX-38, AβX-40, AβX-42, soluble amyloid precursor protein (sAPP)α, and sAPPβ), large fibre axonal degeneration (neurofilament light chain (NFL)), and neuroinflammation (YKL-40) in 245 patients with a variety of dementias and 30 controls. Patients fulfilled consensus criteria for AD (*n* = 156), DLB (*n* = 20), behavioural variant frontotemporal dementia (bvFTD; *n* = 45), progressive non-fluent aphasia (PNFA; *n* = 17), and semantic dementia (SD; *n* = 7); approximately 10% were pathology/genetically confirmed (*n* = 26). Global tests based on generalised least squares regression were used to determine differences between groups. Non-parametric receiver operating characteristic (ROC) curves and area under the curve (AUC) analyses were used to quantify how well each biomarker discriminated AD from each of the other diagnostic groups (or combinations of groups). CSF cut-points for the major biomarkers found to have diagnostic utility were validated using an independent cohort which included causes of AD (*n* = 104), DLB (*n* = 5), bvFTD (*n* = 12), PNFA (*n* = 3), SD (*n* = 9), and controls (*n* = 10).

**Results:**

There were significant global differences in Aβ1–42, T-tau, T-tau/Aβ1–42 ratio, P-tau-181, NFL, AβX-42, AβX-42/X-40 ratio, APPα, and APPβ between groups. At a fixed sensitivity of 85%, AβX-42/X-40 could differentiate AD from controls, bvFTD, and SD with specificities of 93%, 85%, and 100%, respectively; for T-tau/Aβ1–42 these specificities were 83%, 70%, and 86%. AβX-42/X-40 had similar or higher specificity than Aβ1–42. No biomarker or ratio could differentiate AD from DLB or PNFA with specificity > 50%. Similar sensitivities and specificities were found in the independent validation cohort for differentiating AD and other dementias and in a pathology/genetically confirmed sub-cohort.

**Conclusions:**

CSF AβX-42/X-40 and T-tau/Aβ1–42 ratios have utility in distinguishing AD from controls, bvFTD, and SD. None of the biomarkers tested had good specificity at distinguishing AD from DLB or PNFA.

**Electronic supplementary material:**

The online version of this article (10.1186/s13195-018-0361-3) contains supplementary material, which is available to authorized users.

## Background

Cerebrospinal fluid (CSF) biomarkers are increasingly used to support a diagnosis of Alzheimer’s disease (AD). CSF amyloid beta (Aβ)1–42, total tau (T-tau), and phosphorylated tau (P-tau) have utility in differentiating AD from controls and in predicting conversion from mild cognitive impairment (MCI) to AD dementia [1, 2]. Consequently, these measures are included in clinical [3] and research diagnostic criteria [4].

A variety of other CSF measures relevant to neurodegeneration are now available. These include markers of amyloid processing (AβX-38, AβX-40, AβX-42, soluble amyloid precursor protein (sAPP)α, and sAPPβ), large fibre axonal degeneration (neurofilament light chain (NFL)), and neuroinflammation (chitinase-3-like protein 1, also known as YKL-40). The AβX-42/X-40 ratio rather than Aβ1–42 alone may correct for inter-individual differences in amyloid production [5] and may improve clinical diagnostic specificity [6]. Meta-analytical data confirm that YKL-40 and NFL are elevated in clinically diagnosed AD CSF compared with controls [2].

While most prior studies have focussed on distinguishing patients with AD from controls or predicting MCI conversion to AD, a major challenge in clinical practice is to distinguish AD from other neurodegenerative disorders, including frontotemporal dementia (FTD), dementia with Lewy bodies (DLB), semantic dementia (SD), and progressive non-fluent aphasia (PNFA). Here, the role of CSF biomarkers is much less well established.

The principal aims of this study were to determine the diagnostic utility of an extended panel of CSF biomarkers (including two biomarker ratios) both individually and in models incorporating multiple biomarkers to distinguish AD from a range of other primary neurodegenerative dementias in clinical practice, and to validate diagnostic cut-points using a second, independent cohort.

## Methods

The study was conducted in accordance with relevant clinical research regulations, and with ethical approvals in place (Queen Square ethics committee approval reference numbers 13 LO 1155 and 12 LO 1504). Written informed consent was obtained from participants where appropriate.

Two independent cohorts were studied. A test cohort was used to estimate cut-points and to determine the diagnostic utility of each biomarker for differentiating AD from the other groups. A validation cohort was then used to assess the sensitivity and specificity of these cut-points to distinguish AD from all other subjects, from controls, and from other dementias.

### Test cohort

We included individuals referred to the Queen Square Specialist Cognitive Disorders service who had a diagnostic CSF examination between 1 January 2008 and 1 January 2012. Without knowledge of the CSF result, electronic patient records were interrogated to determine the pre-lumbar puncture (LP) diagnosis, most recent clinical diagnosis, time from earliest symptom (reported by individual or their family/caregiver) to LP, mini-mental state examination (MMSE) score at LP, and time from LP to most recent clinical assessment. Consensus criteria were used to classify individuals as: probable AD (including amnestic, logopenic aphasia, and posterior cortical atrophy variants) [3]; DLB [7]; behavioural variant FTD (bvFTD) [8]; PNFA [8]; and SD [8, 9]. The diagnosis was confirmed in 20 cases at autopsy; two patients with AD had *presenilin* 1 mutations, and three cases of BvFTD had C9ORF72 mutations and one a Tau mutation. The pre-LP clinical diagnosis (i.e. without the CSF result) was used for establishing biomarker utility. A second neurologist independently assessed approximately 45% of the cases notes; there was 95.8% diagnostic agreement between raters.

### Validation cohort

All individuals seen in our service who had a diagnostic CSF examination between 16 May 2013 and 16 May 2016 and who fulfilled consensus criteria for a dementia diagnosis (as above) were included. Twelve individuals with an AD diagnosis had an amyloid positron emission tomography (PET) scan, which was positive in all cases.

### Healthy controls

Healthy controls were recruited for research and were usually partners of affected individuals. No control had a memory complaint at recruitment or at 1-year follow-up.

### Sample treatment and analysis

CSF was collected as previously described [10], i.e. by LP between 9 am and 3 pm into a polypropylene vessel, centrifuged, and frozen. Samples were thawed at the bench for 1 h. The volume of CSF differed between individuals; accordingly, not all biomarker measurements were made for all members of the test cohort (see Table 1 for details).

**Table 1:** Test cohort demographic and biomarker data for all diagnostic groups

	AD (*n* = 156)	DLB (*n* = 20)	bvFTD (*n* = 45)	PNFA (*n* = 17)	SD (*n* = 7)	Controls (*n* = 30)
Age at LP (years)	62.5 (57–68)	70.0 (68–75)	61.0 (57–66)	65.0 (61–69)	62.0 (57–68)	63.5 (50–67)
% Male	42.3	75.0	60.0	47.1	71.4	46.7
Symptom onset to LP (months)	36 (24–60) (*n* = 154)	36 (18.5–48)	36 (24–60) (*n* = 44)	36 (24–48)	60 (18–72)	N/A
MMSE	22 (17–25) (*n* = 142)	22 (18–28) (*n* = 15)	24 (18–27) (*n* = 42)	25 (9.5–28) (*n* = 8)	27 (16–27) (*n* = 7)	30 (30–30)
Duration of follow-up (months)	12 (6–24)	11 (4–29.5)	11 (6–23)	12 (4–24)	23 (11–43)	N/A
Aβ1–42 (pg/mL)	310.5 (218.0–451.5)	357.5 (327.0–490.0)	638.0 (396.0–871.0)	440.0 (308.0–696.0)	767.0 (633.0–859.0)	953.0 (771.0–1199.0)
T-tau (pg/mL)	674.5 (430.0–973.5)	338.5 (185.0–489.0)	289.0 (187.0–389.0)	501.0 (367.0–744.0)	319.0 (229.0–458.0)	303.5 (189.0–402.0)
T-tau/Aβ1–42 ratio	2.3 (1.2–3.7) (*n* = 154)	0.8 (0.4–1.5)	0.4 (0.3–0.7) (*n* = 44)	1.1 (0.7–2.1)	0.5 (0.3–0.6)	0.3 (0.2–0.4)
P-tau-181 (pg/L)	86.4 (59.4–111.8) (*n* = 119)	47.1 (38.1–64.3) (*n* = 16)	49.2 (37.0–64.0) (*n* = 39)	62.5 (49.8–100.1) (*n* = 13)	50.9 (25.5–58.6)	47.8 (39.3–65.4) (*n* = 26)
NFL (ng/L)	1191.5 (857.6–1584.0) (*n* = 119)	929.6 (839.9–1650.1) (*n* = 17)	1788.4 (839.9–3334.6) (*n* = 38)	1974.9 (1627.7–3490.5) (*n* = 12)	2400.0 (1687.5–3584.7) (*n* = 6)	649.0 (515.9–849.5)
YKL-40 (ng/mL)	163 (127–194)(*n* = 114)	158 (134–186)(*n* = 16)	163 (135–244)(*n* = 35)	192 (140–207)(*n* = 10)	179 (132–256)(*n* = 5)	111 (93–164)(*n* = 29)
AβX-38 (ng/L)	1462.0 (1101.4–2025.5) (*n* = 117)	1214.2 (840.1–1529.2) (*n* = 15)	1306.0 (1106.2–1658.8) (*n* = 34)	1653.8 (1251.7–2046.7) (*n* = 12)	1751.4 (1442.0–1777.0) (*n* = 5)	2183 (1980.8–3058.6) (*n* = 29)
AβX-40 (ng/L)	3635.1 (2911.0–4584.4) (*n* = 117)	2916.1 (2235.6–3718.2) (*n* = 15)	3439.5 (2714.7–4274.9) (*n* = 34)	3900.6 (3175.7–4355.6) (*n* = 12)	3965.4 (3702.2–4537.6) (*n* = 5)	5478.3 (4888.3–7615.2) (*n* = 29)
AβX-42 (ng/L)	164.6 (109.1–231.6) (*n* = 117)	182.1 (170.9–281.7) (*n* = 15)	284.5 (195.2–369.4) (*n* = 34)	183.0 (117.4–343.6) (*n* = 12)	346.0 (309.9–372.1) (*n* = 5)	592.2 (469.7–749.8) (*n* = 29)
AβX-42/X-40 ratio	0.043 (0.036–0.053) (*n* = 117)	0.055 (0.047–0.089) (*n* = 15)	0.083 (0.072–0.094) (*n* = 34)	0.052 (0.040–0.087) (*n* = 12)	0.087 (0.085–0.093) (*n* = 5)	0.107 (0.092–0.114) (*n* = 29)
APPα (ng/mL)	348.8 (254.9–532.7) (*n* = 119)	218.6 (175.8–368.1) (*n* = 16)	270.7 (164.9–328.7) (*n* = 37)	374.3 (316.1–467.3) (*n* = 12)	379.5 (281.7–479.8) (*n* = 5)	426.4 (322.0–654.5)
APPβ (ng/mL)	202.2 (151.2–325.8) (*n* = 119)	138.0 (115.0–175.2) (*n* = 16)	128.0 (107.4–187.1) (*n* = 37)	220.3 (178.8–298.1) (*n* = 12)	181.9 (171.4–236.4) (*n* = 5)	258.6 (182.0–372.0)

Median and interquartile ranges are shown

Where data were missing, the number of subjects for which data were available is indicated within parentheses

*Aβ* amyloid beta, *AD* Alzheimer’s disease, *APP* amyloid precursor protein, *bvFTD* behavioural variant frontotemporal dementia, *DLB* dementia with Lewy bodies, *LP* lumbar puncture, *PNFA* progressive non-fluent aphasia, *SD* Semantic dementia, *MMSE* mini-mental state examination, *NFL* neurofilament light chain, *P-tau* phosphorylated tau, *T-tau* total tau

Aβ1–42, T-tau, and P-tau assays were performed in batches according to local laboratory standard operating procedures to achieve inter-day coefficients of variation (CV) < 10%. Other assays (AβX-38, AβX-40 and AβX-42, NFL, YKL-40, sAPPα, and sAPPβ) were carried out at a single time point in the Neurochemistry laboratory of the University of Gothenburg by board-certified laboratory technicians. We achieved inter-plate CV of around < 10% for all assays except sAPPα and sAPPβ (details are provided in Additional file 1). The validation cohort were tested at the Institute of Neurology, UCL. Details of the CSF methodology are provided in Additional file 1.

### Statistical analysis

Analyses were carried out using Stata Version 14.1 (Texas, USA). Data distribution was assessed and values outside an assay’s reliable detectable range were assigned maximum/minimum values. Medians and interquartile ranges were used to describe demographic and clinical characteristics and CSF biomarker data by diagnostic group. Missing CSF biomarker values were assumed to be missing completely at random [11], i.e. that the missingness mechanism was unrelated to any covariates relevant to the analysis. CSF biomarkers were compared between diagnostic groups using log-transformed data due to skewed and/or truncated data, and a generalised least squares linear regression model was used (an extension of the *t* test/analysis of variance (ANOVA) model that allows different group-specific residual variances). These global tests for differences between groups were assessed first across all groups including healthy controls, then only in cases with dementia, and finally in cases with dementia also adjusting for age, sex, and disease duration. Post-hoc pairwise comparisons between diagnostic groups were made when the initial (unadjusted) global test across dementia-only groups was statistically significant (*p* < 0.05), and in any biomarker where the unadjusted *p* value was > 0.05 but the adjusted *p* value was < 0.05. For the pairwise comparisons, a conservative Bonferroni-adjusted threshold *p* value for significance (*p* < 0.003) was also used, based on 15 pairwise tests for each biomarker.

Non-parametric receiver operating characteristic (ROC) curves and the area under the curve (AUC) were used to quantify how well each biomarker discriminated between AD and each other diagnostic group (or combinations of groups). The group sizes varied greatly, reflecting the prevalence of these conditions in the population. Assuming that a biomarker is associated with disease, AUC can be considered a simple measure of the probability that a randomly selected case would have a higher biomarker value than a control, assuming higher values are associated with disease (vice versa if lower values are associated with disease) [12].

For the five best-performing (based on AUCs) biomarkers for each of the group comparisons, cut-points and conservative exact binomial confidence intervals were estimated for a set sensitivity of 85%, as suggested by the Reagan consensus report [13], and the associated specificities calculated. For a set sensitivity of 85% (i.e. an 85% probability of a positive test among patients with disease), given that AD is always set as the ‘case’ in any comparison, the optimal cut-point for any specific biomarker is the same regardless of which other diagnostic group is being used as the comparator; it is the specificity that changes for different comparators.

ROC curves from logistic regression models incorporating up to five best-performing biomarkers (based on highest AUC) were used to calculate AUCs where group sizes were sufficiently large (> 10 subjects in each of two groups compared) to avoid over-fitting, with bias corrected bootstrapped confidence intervals for the AUC (2000 replications). The analyses used the ‘leave one out’ approach to address the potential for over-optimistic estimates of AUCs and specificities obtained from these joint models, as this can particularly be an issue when AD cases greatly outnumber the comparator group.

The estimated cut-points of those biomarkers which showed utility in differentiating AD from one or more groups in the test cohort, and for which measures were available, were used to calculate sensitivity and specificity in the validation cohort; due to the small numbers in some diagnostic groups, we only assessed the ability to distinguish AD from controls, from other dementias, and from other dementias and controls combined. Similarly, sensitivity and specificity were calculated in the pathology/genetically confirmed sub-cohort to distinguish AD from other dementias.

## Results

### Subject demographics

We included 418 subjects, 275 in the test and 143 in the validation cohorts. The test cohort comprised 245 patients with dementia (AD (*n* = 156, including 27 posterior cortical atrophy (PCA) and 12 logopenic progressive aphasia (LPA)), DLB (*n* = 20), bvFTD (*n* = 45), PNFA (*n* = 17), and SD (*n* = 7)), and 30 controls. All groups had a similar disease duration (symptom onset to LP) except for the SD group who presented later (Table 1). The DLB group was older than the other disease groups and the proportion of males was higher for DLB and SD than the other groups. Of the 143 individuals in the validation cohort, 104 had AD, 29 had other dementias (5 DLB, 12 bvFTD, 3 PNFA, and 9 SD) and 10 were controls.

### Pathology and genetic confirmation

In total 26 subjects were pathologically or genetically confirmed. Eleven subjects in the test cohort who received a clinical diagnosis of AD (including two with PCA) had a pathological diagnosis of AD at autopsy. None of the subjects diagnosed with AD during life had a non-AD pathological diagnosis. A further two subjects had presenilin 1 mutations known to cause AD. One subject with DLB received a pathological diagnosis of mixed AD/DLB pathology. Five cases with a clinical diagnosis of bvFTD received a pathological diagnosis: one had frontotemporal lobar degeneration with TDP-43 pathology type 3; one had a tauopathy with features compatible with chronic traumatic encephalopathy; one had Pick’s disease; one had FTLD-TDP Type A; and one had mixed AD, Lewy body pathology, and TDP 43 pathology. Four further cases had confirmed genetic bvFTD (three with C9ORF72 mutations and one a Tau mutation). Two patients with PNFA reached autopsy. One had mixed pathology with Pick’s disease, AD pathology, cerebral amyloid angiopathy Lewy body pathology and the other FTLD-TDP Type A pathology. One patient with SD received a pathological diagnosis of FTLD-TDP Type C pathology.

### CSF biomarker concentrations

**Fig. 1:**
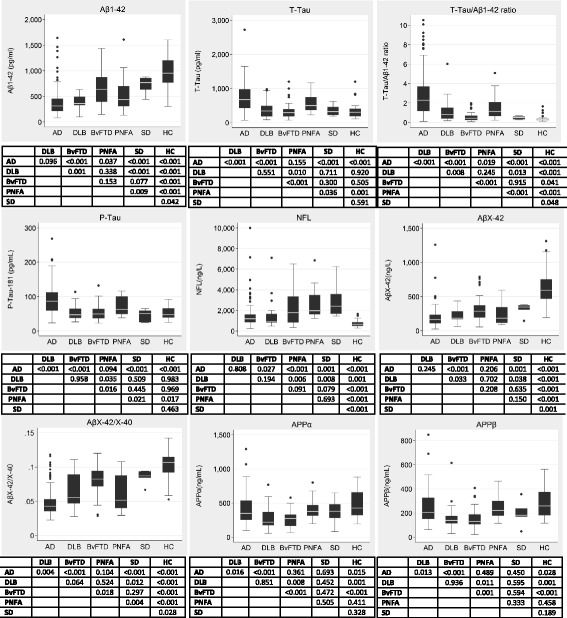
Box-plots and whiskers (25th–75th percentiles) and outliers of measured biomarker concentrations presented by disease group (pre-lumbar puncture diagnosis) and unadjusted pairwise comparisons (*p*-values). X-axis: pre-lumbar puncture diagnosis. Aβ amyloid beta, AD Alzheimer’s disease, APP amyloid precursor protein, bvFTD behavioural variant frontotemporal dementia, DLB Lewy body dementia, HC healthy controls, NFL neurofilament light chain, PNFA progressive non-fluent aphasia, P-tau phosphorylated tau, SD semantic dementia, T-tau total tau

The biomarker profile of each diagnostic group in the test cohort is shown in Table 1 and box-plots are provided in Fig. 1. In the validation cohort data were available for five biomarkers/ratios: Aβ1–42 (*n* = 143); T-tau (*n* = 143); P-tau (*n* = 131); T-tau/Aβ1–42 ratio (*n* = 143); and AβX-42/X-40 ratio (*n* = 140). In the pathology/genetically confirmed sub-cohort, data were available for: Aβ1–42 (*n* = 26); T-tau (*n* = 26); P-tau (*n* = 19); T-tau/Aβ1–42 ratio (*n* = 26); and AβX-42/X-40 ratio (*n* = 17).

Comparisons between the groups based on regression analyses are shown in Table 2. There was a significant difference (*p* < 0.05) between disease groups for all tested biomarkers when controls were included. When excluding the control group this remained the case for nine measures. Additionally, when adjusting for age, sex, and disease duration there was evidence for a difference (*p* = 0.04) between groups for one additional biomarker (YKL-40) whereas no difference had been apparent in the unadjusted analysis (*p* = 0.51).

**Table 2:** Regression analyses comparing biomarkers between all disease groups classified according to pre-lumbar puncture diagnosis, with and without healthy controls

	Global test* including HC (*p* value)	Global test** excluding HC (*p* value)	Adjusted*** global test excluding HC (*p* value)
Aβ1–42	< 0.0001	< 0.0001	< 0.0001
T-tau	< 0.0001	< 0.0001	< 0.0001
T-tau/Aβ1–42 ratio	< 0.0001	< 0.0001	< 0.0001
P-tau-181	< 0.0001	< 0.0001	< 0.0001
NFL	< 0.0001	< 0.0001	< 0.0001
YKL-40	0.0038	0.51	0.04
AβX-38	< 0.0001	0.43	0.17
AβX-40	< 0.0001	0.57	0.30
AβX-42	< 0.0001	0.0001	0.0002
AβX-42/X-40 ratio	< 0.0001	< 0.0001	< 0.0001
APPα	< 0.0001	< 0.0001	0.0001
APPβ	< 0.0001	0.0001	0.0001

Biomarker data are log transformed to achieve normal distribution

*Aβ* amyloid beta, *APP* amyloid precursor protein, *HC* healthy controls, *NFL* neurofilament light chain, *P-tau* phosphorylated tau, *T-tau* total tau

*p < 0.05 provides evidence that the disease groups, including the HC group, do not all have the same mean biomarker value

**As for *, excluding control group

***As for **, also adjusting for age, sex, and time from symptom onset to lumbar puncture

Figure 1 shows pairwise comparisons between diagnostic groups where the (unadjusted) global test across dementia-only groups was statistically significant (unadjusted *p* < 0.05). A summary of where there was evidence of a difference in mean biomarker concentration is shown in Table 3 for each pairwise comparison, both for an unadjusted *p* < 0.05 threshold for significance and a conservative Bonferroni-adjusted *p* < 0.003 threshold.

**Table 3:** Summary of the biomarkers that are significantly different between neurodegenerative disorders

	Aβ1–42	T-tau	T-tau/Aβ1–42	P-tau	NFL	AβX-42	AβX-42/X-40	APPα	APPβ
AD vs DLB		+ ++	+ ++	+ ++			+	+	+
AD vs bvFTD	+ ++	+ ++	+++	+ ++	+	+ ++	+ ++	+ ++	+ ++
AD vs PNFA	+		+		+ ++				
AD vs SD	+ ++	+ ++	+ ++	+ ++	+ ++	+ ++	+ ++		
DLB vs bvFTD	+ ++		+			+			
DLB vs PNFA		+		+	+			+	+
DLB vs SD	+ ++		+		+	+	+		
bvFTD vs PNFA		+ ++	+ ++	+			+	+ ++	+ ++
bvFTD vs SD									
PNFA vs SD	+	+	+ ++	+			+		

Biomarkers with “+” distinguish between groups with *p* < 0.05 from the unadjusted analysis, and “++” distinguish between groups with Bonferroni corrected *p* < 0.003

*Aβ* amyloid beta, *AD* Alzheimer’s disease, *APP* amyloid precursor protein, *bvFTD* behavioural variant frontotemporal dementia, *DLB* dementia with Lewy bodies, *PNFA* progressive non-fluent aphasia, *SD* Semantic dementia, *NFL* neurofilament light chain, *P-tau* phosphorylated tau, *T-tau* total tau

Based on the conservative Bonferroni-adjusted threshold for significance, T-tau/Aβ1–42 ratio, T-tau, and P-tau were significantly elevated in AD compared with each of the other neurodegenerative disorders tested, except PNFA. AβX-42/AβX-40 was significantly lower in the AD cohort than in bvFTD and SD. Aβ1–42 concentrations were lowest in the AD and DLB groups; there was no evidence this biomarker differed between these two disease groups. NFL was significantly higher in all neurodegenerative disorders compared with healthy controls (Fig. 1); concentrations were higher in the SD and PNFA groups compared with the AD group (Table 3). APPα and APPβ were significantly lower in bvFTD compared with AD, PNFA, and healthy controls (Fig. 1).

AβX-38 and AβX-40 concentrations were lower in all neurodegenerative diseases, except SD, compared with controls (*p* < 0.001) but there were no pairwise significant differences between each of the diseases. YKL-40 concentrations were higher across all dementias relative to healthy controls but not between diseases in the unadjusted analyses; after adjusting for age, sex, and time from symptom onset to LP there was evidence of a difference between DLB and bvFTD (*p* = 0.003).

### Diagnostic utility of CSF biomarkers

Cut-points for each biomarker at a pre-determined fixed sensitivity of 85% are shown in Table 4. A summary of the ‘top 5’ biomarkers (by AUC) is given in Table 5, with the highest AUCs varying between 0.79 and 0.95; the specificities are also shown and varied between 24% and 100%.

**Table 4:** Optimal cut-point (95% CI) for AD* at a sensitivity of 85%

Biomarker	Cut-point	95% CI	
		Lower	Upper
Aβ1–42 (pg/mL)	< 529.0	479.0	647.0
T-tau (pg/mL)	> 312.0	261.0	391.0
T-tau/Aβ1–42 ratio	> 0.64	0.52	1.01
P-tau (pg/L)	> 48.9	42.4	58.7
AβX-42/X-40	< 0.060	0.055	0.088
APPβ (ng/mL)	> 136.4	115.3	144.6
NFL (ng/L)	< 1877.0	609.8	3149.6

*For a set sensitivity of 85%, given that AD is always set as the ‘case’ in any comparison, the optimal cut-point for any specific biomarker is the same regardless of which other diagnostic group is being used as the comparator; it is the specificity that changes for different comparators

*Aβ* amyloid beta, *AD* Alzheimer’s disease, *APP* amyloid precursor protein, *CI* confidence interval, *NFL* neurofilament light chain, *P-tau* phosphorylated tau, *T-tau* total tau

**Table 5:** AUC (and 95% CI) and specificity (at a fixed sensitivity of 85%) of the ‘top 5’ biomarkers, comparing AD with other neurodegenerative disorders and controls

Diagnostic groups	Biomarker	AUC (95% CI)	Specificity (%)*
AD vs HC	AβX-42/X-40 ratio	0.95 (0.92–0.99)	93%
	Aβ1–42 (pg/mL)	0.93 (0.88–0.98)	90%
	T-tau/Aβ1–42 ratio	0.93 (0.89–0.97)	83%
	T-tau (pg/mL)	0.81 (0.73–0.90)	53%
	P-tau (pg/L)	0.80 (0.71–0.88)	54%
	All the above	0.91 (0.84–0.95)	88%
AD vs DLB	P-tau (pg/L)	0.79 (0.68–0.90)	50%
	T-tau (pg/mL)	0.78 (0.67–0.88)	50%
	T-tau/Aβ1–42 ratio	0.77 (0.66–0.88)	40%
	AβX-42/X-40 ratio	0.73 (0.59–0.88)	47%
	APPβ (ng/mL)	0.73 (0.58–0.87)	44%
	All the above	0.75 (0.54–0.88)	50%
AD vs bvFTD	T-tau/Aβ1–42 ratio	0.89 (0.85–0.94)	70%
	AβX-42/X-40 ratio	0.86 (0.77–0.94)	85%
	T-tau (pg/mL)	0.83 (0.76–0.90)	64%
	Aβ1–42 (pg/mL)	0.78 (0.70–0.87)	60%
	P-tau (pg/L)	0.78 (0.70–0.86)	46%
	All the above	0.86 (0.78–0.92)	81%
AD vs PNFA^a^	NFL (ng/L)	0.84 (0.76–0.93)	50%
	T-tau/Aβ1–42 ratio	0.67 (0.54–0.80)	24%
	Aβ1–42 (pg/mL)	0.65 (0.50–0.80)	35%
	All the above	0.60 (0.16–0.76)	42%
AD vs SD^b^	AβX-42/X-40 ratio	0.92 (0.86–0.97)	100%
	T-tau/Aβ1–42 ratio	0.91 (0.86–0.96)	86%
	Aβ1–42 (pg/mL)	0.91 (0.84–0.98)	86%
	NFL (ng/L)	0.87 (0.78–0.96)	67%
	P-tau (pg/L)	0.85 (0.75–0.94)	29%
AD vs non-AD dementia	T-tau/Aβ1–42 ratio	0.82 (0.77–0.88)	56%
	AβX-42/X-40 ratio	0.79 (0.72–0.87)	68%
	T-tau (pg/mL)	0.77 (0.71–0.83)	51%
	P-tau (pg/L)	0.76 (0.70–0.83)	41%
	Aβ1–42 (pg/mL)	0.73 (0.67–0.80)	48%
	All the above	0.81 (0.73–0.85)	68%
AD vs all others (including HC)	T-tau/Aβ1–42 ratio	0.85 (0.80–0.90)	63%
	AβX-42/X-40 ratio	0.84 (0.79–0.90)	76%
	T-tau (pg/mL)	0.78 (0.73–0.84)	51%
	Aβ1–42 (pg/mL)	0.78 (0.73–0.84)	59%
	P-tau (pg/L)	0.77 (0.71–0.83)	45%
	All the above	0.84 (0.79–0.90)	75%

*Aβ* amyloid beta, *AD* Alzheimer’s disease, *APP* amyloid precursor protein, *AUC* area under the curve, *bvFTD* behavioural variant frontotemporal dementia, *CI* confidence interval, *DLB* dementia with Lewy bodies, *HC* healthy controls, *PNFA* progressive non-fluent aphasia, *SD* Semantic dementia, *NFL* neurofilament light chain, *P-tau* phosphorylated tau, *T-tau* total tau

^a^Only three biomarkers were found to be significant, see Table 3

^b^There is no joint model for AD vs SD because *n* < 10 for SD

Table 5 also shows the results from incorporating the best-performing biomarkers into a single model for each of the comparisons of AD against other groups. There was no suggestion that including more than one biomarker usefully improved AUC or specificity when compared to the single biomarker with highest AUC or specificity, respectively.

### Validation

In the validation cohort we calculated sensitivity and specificity for Aβ1–42, T-tau, P-tau, T-tau/Aβ1–42, and AβX-42/X-40 using the optimal cut-points determined in the test cohort that provided a sensitivity of 85% (Additional file 2: Table S1). Sensitivities were very consistent with the 85%, ranging from 83 to 88% for all biomarkers compared between all groups except for Aβ1–42 where the sensitivity was lower (71%). We also calculated sensitivities and specificities of these biomarkers for the pathologically or genetically defined cases (*n* = 26) (Additional file 2: Table S1), finding superior sensitivities (83–100%) and broadly comparable specificities given the smaller sample sizes and missing values for some biomarkers.

## Discussion

In this single centre, primarily clinic-based study we show that some biomarkers with proven ability to distinguish AD from healthy controls [2] also have utility for differentiating AD from other neurodegenerative dementias in clinical practice. In particular, T-tau/Aβ1–42 and AβX-42/X-40 ratios combine high sensitivity (85%) and good specificity (> 70%) for distinguishing AD not only from controls but also from SD and bvFTD; Aβ1–42 performed similarly well for distinguishing AD from controls and SD. In contrast, none of the biomarkers, or models with multiple biomarkers, could reliably differentiate AD from DLB or PNFA with high specificity.

The cut-points we generated are similar to those found in other studies. For differentiating AD subjects from healthy controls we found broad agreement with those reported in previous studies [14] for Aβ1–42, T-tau/Aβ1–42, and AβX-42/X-40. The exception was P-tau, where our cut-point (48.9 pg/mL) was lower than that quoted by the kit manufacturer (61 pg/mL) [15]. This may reflect our choice of a set sensitivity of 85% (resulting in a specificity of 54%) compared with the manufacturer’s 80% (with a specificity of 87%).

Overall, we found no evidence that models incorporating multiple biomarkers (or simple ratios) materially improved AUC or specificity compared to the best-performing single biomarker (or ratio) with highest AUC or specificity, respectively. Specifically, for AD vs healthy controls we were able to achieve good sensitivity and specificity using Aβ1–42, T-tau/Aβ1–42, and AβX-42/X-40 without using complex models of multiple biomarkers or formulae that have been proposed in other studies [16, 17].

It was possible to differentiate AD from SD or bvFTD with good sensitivity and specificity particularly using AβX-42/X-40. While the 100% specificity for AβX-42/X-40 to distinguish AD from SD is inevitably influenced by the small SD sample size, the generally high specificities are likely to reflect that SD is very pathologically homogeneous, typically being underpinned by TDP 43 type C pathology [18, 19] as was the case in the one SD case in this cohort who came to autopsy. Using AβX-42/X-40, the specificity for AD versus bvFTD was still high (85%) despite the fact that bvFTD can sometimes be caused by AD pathology, or have co-existent AD pathology [19].

We found that no single or ratio of CSF biomarkers achieved useful specificity for distinguishing AD from DLB [20, 21]. P-tau and T-tau were the best performing biomarkers but, consistent with a previous meta-analysis [22], they were not diagnostically useful, achieving specificities of only approximately 50%. This is likely to reflect that AD pathology is very common in pathologically confirmed DLB [23], as was seen in the one subject in this cohort with clinically diagnosed DLB who had mixed AD/DLB pathology at autopsy. Improving specificity is therefore likely to require a positive biomarker for DLB pathology, e.g. a reliable marker of alpha-synuclein inclusions. An enzyme-linked immunosorbent assay (ELISA) biomarker for DLB pathology has slightly improved the diagnostic utility of CSF biomarkers for differentiating AD from DLB [24]; more recently, a real-time quaking induced conversion assay (RT-QUIC) showed significant promise as a highly specific test for DLB pathology [25].

None of the biomarkers was useful for differentiating AD from PNFA; the best performing measure was NFL, which achieved a specificity of only 50%. PNFA is classically considered within the FTD spectrum, but 10–30% of cases have AD pathology at autopsy [26, 27]. In this cohort two PNFA case had had an autopsy, where mixed pathology (Pick’s disease, AD, cerebral amyloid angiopathy, and Lewy Body pathology) and FTLD TDP 43 pathology were found. The relatively poor specificity for any CSF biomarker in this group is likely therefore to reflect cases of PNFA due to AD, and PNFA with mixed AD pathology, and emphasizes the need for pathology-specific biomarkers for the non-AD dementias.

While T-tau/Aβ1–42 ratio performed well in several of the disease group comparisons, neither T-tau nor P-tau was diagnostically useful alone, conferring specificities of at most 64%. CSF Aβ1–42 alone was relatively poor at distinguishing AD from other neurodegenerative disorders (except for SD), in line with other studies [22]. Specificity was, however, consistently improved using the AβX-42/X-40 ratio [28–30]. AβX-40 is the most abundant soluble Aβ peptide and less likely than Aβ1–42 to aggregate, and thus incorporating both in a ratio may account for inter-individual physiological differences in amyloid processing [31]. AβX-42/X-40 ratio performed at least as well as T-tau/Aβ1–42 ratio; adding T-tau to AβX-42/X-40 did not improve specificity, suggesting that the AβX-42/X-40 ratio alone may a reliable means of identifying brain amyloid deposition.

While the focus of the study was on differentiating AD from other dementias, a number of potentially interesting findings emerge from some of the more novel biomarkers. Our finding that NFL concentration was highest in SD is consistent with a number of previous studies [32–34]. NFL is thought to be a marker of large axonal neurodegeneration [35] and is elevated in a number of non-AD diseases [36–38], particularly FTD and motor neurone disease [39]. We found that the concentration of YKL-40 was elevated in AD compared to controls, in keeping with prior studies [2, 40]. We did not find either APPα or APPβ to be useful in differentiating AD from controls.

This study has a number of caveats. We used clinical diagnosis based on a blinded independent assessment using contemporary clinical criteria to establish the diagnosis, rather than post-mortem confirmation of underlying pathology or pathologies. Very few CSF studies in dementia have pathological confirmation of diagnosis, and this is therefore a limitation of most work in the literature. However, we were able to confirm a definite pathological or genetic diagnosis in 26/245 subjects with dementia in the test cohort. In cases fulfilling clinical criteria for AD, approximately 10% had either pathological confirmation, genetic confirmation, or supportive amyloid imaging, with no false positive diagnoses. Similarly, bvFTD and SD diagnoses were supported by pathological confirmation in approximately 10% of cases with all having FTD pathology or mixed FTD/AD pathology.

There is not perfect concordance between clinical diagnosis and underlying pathology, and this varies considerably depending on the clinical syndrome. In patients diagnosed with probable AD, the sensitivity and specificity for underlying AD pathology are in the order of approximately 75% and 60%, respectively [41]. AD pathology is found in approximately 55% of cases of DLB [42], approximately 40% of PNFA cases [43], 5–6% of bvFTD [44], and between 0 and 15% of SD cases [19, 45, 46]. The results in this study are broadly consistent with these figures; indeed, the best specificity found for each group is strikingly similar to the proportion who would be expected not to have AD pathology at post mortem (SD 100%, bvFTD 85%, PNFA 50%, DLB 50%). This is therefore consistent with our interpretation that current biomarkers are good at distinguishing AD from syndromes that are not usually caused by AD (e.g. SD and bvFTD) but not from those commonly caused by AD (PNFA) or where there is AD co-pathology (DLB).

The number of samples in some groups was comparatively small, particularly in the rarer clinical syndromes, but are likely to represent the proportion of patients who might undergo diagnostic CSF examination. There is no optimal means of determining biomarker cut-points [12], but we used a consistent and recommended method of fixing sensitivity at 85%. There was variability in the inter-plate variability depending on the analyte measured. While most assays achieved inter-day and inter-plate variability of < 10%, we acknowledge that the inter-plate CV for the APP ELISA assays were > 10% and results should be interpreted with caution. Finally, while we used an extended CSF panel, this was not comprehensive and did not for example include neurogranin, which may have good specificity for AD [47].

## Conclusions

Biomarkers in routine clinical use (particularly AβX-42/X-40 and T-tau/Aβ1–42 ratios) not only have utility in distinguishing AD from controls, but also from bvFTD and SD. These measures, and the other biomarkers tested, have less utility in differentiating AD from DLB and PNFA, likely reflecting varying degrees of AD (amyloid) pathology in these conditions. This study provides an evidence base for the use of CSF biomarkers for the differential diagnosis of AD, highlights the potential utility of the AβX-42/X-40 ratio, and shows that novel biomarkers specific for other non-AD disorders are required.

## Additional files

Additional file 1: CSF assay methodology. (DOCX 17 kb)

Additional file 2 **Table S1.** Diagnostic accuracy of Aβ1–42, T-tau, T-tau/Aβ1–42 ratio, P-tau, and AβX-42/X-40 ratio in test and validation cohorts based on pre-LP diagnostic classification and diagnostic accuracy in the pathologically or genetically defined sub-cohort. AD Alzheimer’s disease, DLB dementia with Lewy bodies, bvFTD behavioural variant frontotemporal dementia, PNFA progressive non-fluent aphasia, SD Semantic dementia, HC healthy control. (DOCX 20 kb)

## Abbreviations

AD: Alzheimer’s disease
AUC: Area under the curve
Aβ: Amyloid beta
bvFTD: Behavioural variant frontotemporal dementia
CSF: Cerebrospinal fluid
CV: Coefficients of variation
DLB: Dementia with Lewy bodies
FTD: Frontotemporal dementia
LP: Lumbar puncture
MCI: Mild cognitive impairment
MMSE: Mini-mental state examination
NFL: Neurofilament light chain
PCA: Posterior cortical atrophy
PNFA: Progressive non-fluent aphasia
P-tau: Phosphorylated tau
ROC: Receiver operating characteristic
sAPP: Soluble amyloid precursor protein
SD: Semantic dementia; T-tau, Total tau
