# Supplementary material for: Cerebrospinal fluid in the differential diagnosis of Alzheimer’s disease: clinical utility of an extended panel of biomarkers in a specialist cognitive clinic
Source: Alzheimers Res Ther. 2018 Mar 20;10:32. doi: 10.1186/s13195-018-0361-3 (PMC5861624; doi:10.1186/s13195-018-0361-3)
Supplement: Supplementary file 1 — CSF assay methodology. (DOCX 17 kb) [file 13195_2018_361_MOESM1_ESM.docx]

**Supplementary material – CSF assay methodology**

Total tau (T-tau), phosphorylated tau (P-tau) and β-amyloid 1-42 (Aβ1-42) were analyzed using INNOTEST enzyme-linked immunosorbent assays (ELISAs (Fujirebio Europe N.V., Gent, Belgium). Other markers of amyloid processing were measured using the MSD Aβ Triplex assay (Meso Scale Discovery, Rockville, MD), a multiplexed method in which C-terminally specific antibodies are used selectively to capture Aβ forms ending at amino acids 38, 40 and 42, respectively, which are then quantified using the 6E10 detector antibody. This assay is thus not specific to the 1st amino acid of the Aβ peptides (the epitope of 6E10 lies within amino acids 3 to 8 in the Aβ sequence) and the measured Aβ isoforms are therefore called AβX-38, AβX-40 and AβX-42. Neurofilament light chain (NFL) concentrations were determined using the NF-light method (UmanDiagnostics, Umeå Sweden); YKL-40, also known as chitinase-3-like protein 1 (CHI3L1), was measured using the Human Chitinase 3-like 1 Quantikine ELISA Kit (R&D systems, Minneapolis, MN). Amyloid precursor protein soluble metabolites α and β (sAPPα, sAPPβ) were measured using a commercial duplex immunoassay with electrochemiluminescence detection (Meso Scale Discovery, Rockville, MD). Inter-plate co-efficients of variation for internal standards (pooled AD CSF) were: YKL-40: 9.59 %; NFL: 7.72%; sAPPα: 23.03%; sAPPβ: 28.56%; AβX-38: 5.52%; AβX-40: 7.57%; AβX-42: 10.17%.
