# Supplementary material for: Cerebrospinal fluid in the differential diagnosis of Alzheimer’s disease: clinical utility of an extended panel of biomarkers in a specialist cognitive clinic
Source: Alzheimers Res Ther. 2018 Mar 20;10:32. doi: 10.1186/s13195-018-0361-3 (PMC5861624; doi:10.1186/s13195-018-0361-3)
Supplement: Supplementary file 2 — Table S1. Diagnostic accuracy of Aβ1–42, T-tau, T-tau/Aβ1–42 ratio, P-tau, and AβX-42/X-40 ratio in test and validation cohorts based on pre-LP diagnostic classification and diagnostic accuracy in the pathologically or genetically defined sub-cohort. AD Alzheimer’s disease, DLB dementia with Lewy bodies, bvFTD behavioural variant frontotemporal dementia, PNFA progressive non-fluent aphasia, SD Semantic dementia, HC healthy control. (DOCX 20 kb) [file 13195_2018_361_MOESM2_ESM.docx]

**Supplementary Table S1** Diagnostic accuracy of Aβ1-42, T-tau, T-tau/Aβ1-42 ratio, P-tau and AβX-42/X-40 ratio in test and validation cohorts based on pre-LP diagnostic classification and diagnostic accuracy in the pathologically or genetically defined sub-cohort. AD: Alzheimer’s disease; DLB: dementia with Lewy bodies; bvFTD: behavioural variant frontotemporal dementia; PNFA: progressive non-fluent aphasia; SD: Semantic dementia; HC: healthy control.

|  |  | **Test cohort (used to estimate the cut-point)(data from tables 3 and 4)**  **(n=275)** | | **Validation cohort**  **(using test cohort cut-point) (n=143)** | | **Pathologically or genetically confirmed sub-cohort (n=26)** | |
| --- | --- | --- | --- | --- | --- | --- | --- |
| **Diagnostic Groups compared** | **Biomarker** | **Optimal cut-point at 85% sensitivity** | **Specificity** | **Sensitivity** | **Specificity** | **Sensitivity** | **Specificity** |
| **AD vs HC** | AβX-42/X-40 | <0.060 | 93% | 82% | 80% |  |  |
|  | Aβ1-42 (pg/mL) | <529.0 | 90% | 71% | 80% |  |  |
|  | T-tau/Aβ1-42 | >0.64 | 83% | 88% | 89% |  |  |
|  | T-tau (pg/mL) | >312.0 | 53% | 87% | 78% |  |  |
|  | P-tau (pg/L) | >48.9 | 54% | 83% | 78% |  |  |
| **AD vs**  **non-AD dementia** | T-tau/Aβ1-42 | >0.64 | 56% | 88% | 76% | 100% | 60% |
|  | AβX-42/X-40 | <0.060 | 68% | 82% | 74% | 92% | 100% |
|  | T-tau (pg/mL) | >312.0 | 51% | 87% | 53% | 94% | 60% |
|  | P-tau (pg/L) | >48.9 | 41% | 83% | 70% | 83% | 43% |
|  | Aβ1-42 (pg/mL) | <529.0 | 48% | 71% | 77% | 88% | 60% |
| **AD vs all (inc. HC)** | T-tau/Aβ1-42 | >0.64 | 63% | 88% | 76% |  |  |
|  | AβX-42/X-40 | <0.060 | 76% | 83% | 74% |  |  |
|  | T-tau (pg/mL) | >312.0 | 51% | 87% | 50% |  |  |
|  | Aβ1-42 (pg/mL) | <529.0 | 59% | 71% | 77% |  |  |
|  | P-tau (pg/L) | >48.9 | 45% | 83% | 70% |  |  |
